# Clinical characteristics and severity classification in mixed connective tissue disease: a nationwide retrospective study

**DOI:** 10.1093/rheumatology/keag577

**Published:** 2026-10-15

**Authors:** Satoshi Kubo, Yurie Kanda, Hideto Kameda, Masataka Kuwana, Hidehiko Narazaki, Takao Fujii, Keishi Fujio, Yoshinao Muro, Konomi Akamatsu, Koichiro Ohmura, Takehisa Ogura, Yuichiro Shirai, Yujiro Tanabe, Ran Nakashima, Yumi Tsuchida, Shingo Nakayamada, Hisanori Hasegawa, Shintaro Hirata, Shusaku Fukaya, Takashi Kato, Hidekata Yasuoka, Yuta Yamashita, Michihito Kono, Tatsuya Atsumi, Yoshiya Tanaka

**Affiliations:** The First Department of Internal Medicine, University of Occupational and Environmental Health, Kitakyushu, Japan; The First Department of Internal Medicine, University of Occupational and Environmental Health, Kitakyushu, Japan; Division of Rheumatology, Toho University, Tokyo, Japan; Department of Allergy and Rheumatology, Nippon Medical School Graduate School of Medicine, Tokyo, Japan; Department of Pediatrics, Nippon Medical School, Tokyo, Japan; Department of Rheumatology and Clinical Immunology, Wakayama Medical University, Wakayama, Japan; Department of Allergy and Rheumatology, The University of Tokyo, Tokyo, Japan; Department of Dermatology, Fujita Health University, Toyoake, Japan; Division of Rheumatology, Department of Internal Medicine, Fujita Health University, Toyoake, Japan; Department of Rheumatology, Kobe City Medical Center General Hospital, Kobe, Japan; Division of Rheumatology, Toho University, Tokyo, Japan; Department of Allergy and Rheumatology, Nippon Medical School Graduate School of Medicine, Tokyo, Japan; Department of Pediatrics, Nippon Medical School, Tokyo, Japan; Department of Rheumatology and Clinical Immunology, Graduate School of Medicine, Kyoto University, Kyoto, Japan; Department of Allergy and Rheumatology, The University of Tokyo, Tokyo, Japan; The First Department of Internal Medicine, University of Occupational and Environmental Health, Kitakyushu, Japan; Office of Global Affairs, Institute of Science Tokyo, Tokyo, Japan; Department of Clinical Immunology and Rheumatology, Hiroshima University, Hiroshima, Japan; Division of Rheumatology, Department of Internal Medicine, Fujita Health University, Toyoake, Japan; Department of Rheumatology and Clinical Immunology, Wakayama Medical University, Wakayama, Japan; Division of Rheumatology, Department of Internal Medicine, Fujita Health University, Toyoake, Japan; Department of Dermatology, Nagoya University Graduate School of Medicine, Nagoya, Japan; Department of Rheumatology, Endocrinology and Nephrology, Faculty of Medicine, Hokkaido University, Sapporo, Japan; Department of Rheumatology, Endocrinology and Nephrology, Faculty of Medicine, Hokkaido University, Sapporo, Japan; The First Department of Internal Medicine, University of Occupational and Environmental Health, Kitakyushu, Japan; Department of Molecular Targeted Therapeutics, University of Occupational and Environmental Health, Kitakyushu, Japan

**Keywords:** mixed connective tissue disease, anti-U1-RNP antibody, severity, phenotype, nationwide, cohort

## Abstract

**Objectives:**

To characterize clinical manifestations, major organ involvement, real-world treatment patterns and factors associated with severity classification in Japanese patients with mixed connective tissue disease (MCTD), aiming to clarify its heterogeneous clinical spectrum.

**Methods:**

This nationwide retrospective multicentre study analyzed clinical data from 249 patients who strictly fulfilled the 2019 Japanese Ministry of Health, Labour and Welfare diagnostic criteria for MCTD, derived from an initial real-world registry of 311 clinically diagnosed patients. Clinical features, autoantibodies and therapeutic regimens were summarized and compared between patients with severe vs nonsevere disease.

**Results:**

The cohort was 93.1% female (median onset age 40.6 years). Common findings, specifically Raynaud’s phenomenon and/or swelling of the fingers or dorsum of the hands, were present in 100%. Phenotypically, an overlap of systemic lupus erythematosus (SLE)-like and systemic sclerosis (SSc)-like features was most frequent (56.0%), while 32.0% had manifestations across all three phenotypic domains (SLE-, SSc- and IIM-like). Major organ involvement occurred in 32.9% of patients, including pulmonary arterial hypertension in 12.8%. Approximately 28.7% of evaluable patients were classified as severe. Off-scale-high anti-U1-RNP antibody status was not associated with severity classification (*P* = 0.492) or with broad phenotypic categories. Glucocorticoids and immunosuppressive agents were used in 66.7% and 56.5% of the patients, respectively.

**Conclusions:**

Mixed connective tissue disease exhibits marked clinical heterogeneity. Severity classification was not associated with off-scale-high anti-U1-RNP antibody status or broad phenotypic categories. These findings support rigorous cardiopulmonary screening and management tailored to the dominant clinical phenotype.

Rheumatology key messages

- Severity classification was not associated with off-scale-high anti-U1-RNP antibody status or broad phenotypic categories (SLE-, SSc- or IIM-like).
- Phenotypically, 56.0% of patients exhibit SLE and SSc overlaps in this cohort.
- Systematic cardiopulmonary screening remains important because major organ involvement was common in this cohort.

## Introduction

First proposed by Sharp *et al.* in 1972, mixed connective tissue disease (MCTD) is a systemic autoimmune rheumatic disease characterized by anti-U1 ribonucleoprotein (U1-RNP) antibodies together with a constellation of clinical features of systemic lupus erythematosus (SLE), systemic sclerosis (SSc) and idiopathic inflammatory myopathies (IIM) [1]. Following its initial proposal, there has been much debate about the distinction of MCTD from undifferentiated connective tissue disease (UCTD) or overlap syndrome. Subsequent studies have consistently identified a reproducible clinical phenotype defined by Raynaud’s phenomenon and swollen fingers or dorsum of hands, together with characteristic complications such as a high prevalence of pulmonary arterial hypertension (PAH) and trigeminal neuropathy [2–4]. Consequently, MCTD has come to be regarded as a distinct disease entity rather than a subset of UCTD. This concept was further supported by a recent large-scale, multicentre French cohort study by Chevalier *et al.* (2024), which confirmed that MCTD remains a discrete clinical entity distinct from other connective tissue diseases even over long-term follow-up [5]. Despite the established disease concept, the clinical spectrum of MCTD remains heterogeneous, ranging from mild manifestations such as Raynaud’s phenomenon to life-threatening complications, including PAH and progressive interstitial lung disease (ILD), which critically influence prognosis [6, 7]. This heterogeneity, together with unclear distinction from other connective tissue diseases, complicates the standardization of diagnostic approaches, the stratification of disease severity and the development of evidence-based treatment strategies.

Historically, diagnostic and classification criteria for MCTD have been based on the frameworks proposed by Sharp, Alarcón-Segovia, Kahn, Kasukawa and others [8, 9]. In Japan, diagnostic criteria developed by a Japanese Ministry of Health, Labour and Welfare (MHLW) research group were revised in 2004 and 2019 and are now widely applied in both clinical practice and research [10, 11]. In real-world settings, however, some patients receive a clinical diagnosis of MCTD despite not fully fulfilling these contemporary criteria, often due to incomplete testing, missing longitudinal information or discrepancies between symptom onset and documentation [12]. Accordingly, to improve diagnostic homogeneity, the present primary analysis was restricted to patients who strictly fulfilled the 2019 MHLW diagnostic criteria. Moreover, although glucocorticoids and conventional immunosuppressants remain the mainstay of therapy for MCTD, high-quality, disease-specific evidence guiding drug selection, dosing and tapering is still lacking [13]. Clarifying the clinical phenotype, severity profile and real-world treatment patterns of patients who strictly satisfy contemporary MCTD criteria is therefore essential to improving the quality and consistency of care.

Accordingly, we identified 311 patients with a clinical diagnosis of MCTD from a nationwide retrospective multicentre cohort in Japan and restricted the primary analysis to 249 patients who strictly fulfilled the 2019 MHLW diagnostic criteria. We systematically characterized their clinical and immunological profiles, examined the prevalence and patterns of SLE-, SSc- and IIM-like manifestations, and investigated major organ involvement and real-world treatment patterns. We also examined baseline factors associated with severity classification in this strictly defined MCTD cohort.

## Methods

### Study design and population

This study was a retrospective observational analysis of patients with MCTD. We included 311 patients who were clinically diagnosed with MCTD and managed at the University of Occupational and Environmental Health, Japan, Toho University, The University of Tokyo, Kobe City Medical Center General Hospital, Hiroshima University, Kyoto University, Institute of Science Tokyo and Nagoya University. Among them, we restricted our primary analysis to 249 patients who strictly fulfilled the 2019 diagnostic criteria proposed by the Research Committee on Autoimmune Diseases of the MHLW of Japan [10, 11]. The diagnostic criteria were officially described according to the Act on Medical Care for Patients with Intractable Diseases [14]. This study was approved by the ethics review board of the University of Occupational and Environmental Health, Japan (UOEH CRB24-056). This study was conducted in accordance with the Declaration of Helsinki. Informed consent was obtained from patients, or the requirement was waived in accordance with institutional regulations, utilizing anonymized data. The transparency of this study complies with the STROBE statement (Supplementary Data S1).

### Data collection and clinical assessments

Demographic and clinical data were collected from standardized clinical survey forms. We analyzed the clinical signs and laboratory data, including autoantibody profiles and organ involvement assessed at the time of diagnosis. The collected variables included: (1) Patient characteristics: age at onset, sex and family history. (2) Clinical manifestations: presence of common findings (Raynaud’s phenomenon or swelling of the fingers or dorsum of the hands) and mixed findings characteristic of SLE, SSc and IIM. Specific symptoms assessed included polyarthritis, lymphadenopathy, facial erythema, pericarditis/pleuritis, sclerodactyly, interstitial lung disease (ILD), oesophageal dysmotility, muscle weakness, myogenic enzyme elevation and electromyographic abnormalities. (3) Organ involvement: PAH was diagnosed according to the prevailing haemodynamic criteria at the time of clinical assessment and was principally confirmed by right-heart catheterization. Under the historical definition, PAH was characterized by a resting mPAP ≥25 mmHg, PAWP ≤15 mmHg and PVR >3 WU [15]. Because this was a retrospective study using historical registry data, complete haemodynamic variables required to retrospectively apply the current definition of PAH were not uniformly available. ILD was defined on the basis of high-resolution computed tomography (HRCT) abnormalities consistent with ILD as recorded in routine clinical practice by the attending rheumatologists. In this study, the severity and progression of ILD were categorized based on the severity classification criteria for MCTD established by the Japan Research Committee of the MHLW. Advanced ILD was defined as the presence of ILD accompanied by exertional dyspnoea, palpitations or persistent cough. Rapidly Progressive ILD was classified according to the MHLW severity criteria, which define this condition by rapidly progressive respiratory symptoms, including dyspnoea and cough. The MHLW criteria do not specify an explicit temporal threshold for ‘rapidly progressive’. Therefore, this classification was based on the clinical assessment documented by the treating physician. (4) Laboratory findings: complete blood counts (white blood cell count, platelet count) and immunological profiles, including anti-U1-RNP antibody titres and other autoantibodies (anti-dsDNA, anti-Sm, anti-topoisomerase I, anti-RNA polymerase III, anti-aminoacyl tRNA synthetase [ARS], including anti-Jo-1, and anti-CCP antibodies). Leukopoenia was defined as a white blood cell count <4000/µl, and thrombocytopenia was defined as a platelet count <100,000/µl. Anti-U1-RNP antibodies were detected using double immunodiffusion (DID), enzyme-linked immunosorbent assay (ELISA) or chemiluminescent enzyme immunoassay (CLEIA). The measurement method for anti-U1-RNP antibodies transitioned from DID or ELISA to CLEIA around 2013–15, and CLEIA was used exclusively for all assessments thereafter. Other autoantibodies were measured using standardized commercial immunoassays (ELISA or CLEIA) approved for *in vitro* diagnostic use under the Japanese national health insurance system. Because anti-U1-RNP antibody assays differed across institutions and calendar periods, absolute quantitative values were not pooled directly across centres. For analyses of disease severity, antibody titres were therefore harmonized as off-scale high vs not off-scale high, according to the upper limit of each assay used at each institution. (5) Treatment patterns: use of glucocorticoids (GC), GC pulse therapy, immunomodulator/immunosuppressants (e.g. hydroxychloroquine, tacrolimus, methotrexate and cyclophosphamide), biologics and PAH-targeted vasodilators. For analysis of clinical characteristics, organ involvement and treatment patterns, proportions were calculated using the number of patients with available data as the denominator, excluding missing values to reflect distributions among assessed patients. In contrast, for evaluation of diagnostic criteria fulfilment, we applied a strict approach. Patients with missing data required to adjudicate specific criteria components were considered not to meet those components, meaning missing data were treated as absent.

### Diagnostic criteria and disease severity

The fulfilment of the MHLW diagnostic criteria was retrospectively verified for each patient. The criteria require the presence of: (1) at least one common finding, (2) positive anti-U1-RNP antibody and (3) either one or more characteristic organ involvements or mixed findings from at least two of the three disease categories (SLE, SSc and IIM). Disease severity was classified into three grades (Severe, Moderate and Mild) according to the specific disease severity classification for MCTD defined by the MHLW (Supplementary Table S1). In Japan, this severity classification is routinely used as part of the eligibility assessment for public medical expense subsidies for MCTD as a designated intractable disease. Some components require clinical judgement by the treating physician because explicit quantitative thresholds are not specified for all manifestations. For the analysis of factors associated with disease severity, patients were categorized into two groups: the Severe group (Grade 1) and the Moderate/Mild group (Grade 2 and Grade 3). Unless otherwise specified, all descriptive analyses, severity analyses and treatment analyses in the main manuscript were performed in the 249 patients who fulfilled the 2019 MHLW diagnostic criteria. As an additional analysis, the Kasukawa diagnostic criteria were retrospectively applied to the broader clinically diagnosed cohort of 311 patients using the same strict approach to missing data, whereby missing criteria components were treated as absent [10, 16]. Concordance between the 2019 MHLW and Kasukawa criteria was evaluated using overall agreement and Cohen’s *κ*, with the 95% confidence interval for *κ* estimated by nonparametric bootstrap resampling with 100,000 iterations.

### Statistical analysis

All statistical analyses were performed using R statistical software, version 4.5.1. Percentages were calculated based on the number of patients with available data for each variable, excluding missing values from the denominator. Continuous variables are presented as medians with interquartile ranges (IQR) due to non-normal distributions, while categorical variables are presented as numbers and percentages. To identify baseline factors associated with severity classification, we compared demographic, clinical and serological characteristics between the Severe group and the Moderate/Mild group. Differences in continuous variables (e.g. age at onset) were analyzed using the Mann–Whitney *U* test (Wilcoxon rank sum test). Differences in categorical variables (e.g. sex, presence of specific clinical symptoms and autoantibody positivity) were analyzed using the χ^2^ test or Fisher’s exact test, as appropriate. For the exploratory analysis of factors associated with severe classification, manifestations that directly define the severe category, including PAH, advanced or rapidly progressive ILD and cytopenias, were excluded from the predictor analysis to avoid tautological associations. These manifestations were not excluded when assigning the MHLW severity grade itself. Two-sided *P*-values < 0.05 were considered statistically significant. As a sensitivity analysis, multivariable logistic regression was performed with severe disease classification as the dependent variable and anti-U1-RNP antibody off-scale high titre, age at onset, sex, polyarthritis, Raynaud’s phenomenon and sclerodactyly as explanatory variables.

## Results

### Patient characteristics

Of the 311 patients with a clinical diagnosis of MCTD, 249 strictly fulfilled the 2019 MHLW diagnostic criteria and were included in the primary analysis (Table 1). Of the 245 patients with evaluable sex data, 228 (93.1%) were women, and the median age at onset was 40.6 years (IQR 28.5–56.6). A family history of MCTD was present in 17 of 207 evaluable patients (8.2%). By definition, all included patients fulfilled the common findings criterion and were positive for anti-U1-RNP antibodies. Among 237 evaluable patients, Raynaud’s phenomenon was observed in 223 (94.1%). Regarding mixed features, SLE-like phenotypes were present in 232 of 249 patients (93.2%), SSc-like manifestations in 218 of 249 (87.6%) and IIM-like manifestations in 106 of 249 (42.6%). Among SLE-like manifestations, polyarthritis was observed in 168 of 245 patients (68.6%), whereas among SSc-like manifestations, sclerodactyly was present in 173 of 245 (70.6%). Muscle weakness was documented in 57 of 245 patients (23.3%). In terms of characteristic organ involvement, pulmonary arterial hypertension was found in 31 of 243 patients (12.8%), aseptic meningitis in 12 of 244 (4.9%) and trigeminal neuropathy in 10 of 240 (4.2%). In the broader clinically diagnosed cohort (*n* = 311), demographic and clinical features were largely consistent with the strictly defined cohort (Supplementary Table S2). In the additional concordance analysis, 238 of 249 patients (95.6%) who fulfilled the 2019 MHLW criteria also fulfilled the Kasukawa criteria. Overall agreement between the two criteria sets in the broader cohort of 311 patients was 96.1%, with a Cohen’s *κ* of 0.886 (95% CI 0.819–0.944). Of the 11 patients who fulfilled the MHLW criteria but not the Kasukawa criteria, all met the characteristic organ involvement pathway, comprising PAH in six patients, aseptic meningitis in four and trigeminal neuropathy in one (Supplementary Table S3).

**Table 1:** Demographic characteristics and clinical manifestations of patients meeting the 2019 MHLW MCTD diagnostic criteria.

Variables	*n*/*N* (%)
**Demographics**	
Female sex	228/245 (93.1)
Age at onset, years	40.6 [28.5–56.6]
Family history of MCTD	17/207 (8.2)
Common findings	
Raynaud’s phenomenon	223/237 (94.1)
**Immunological findings**	
Anti-U1-RNP antibody positive	249/249 (100.0)
**Mixed findings (organ involvement)**	
SLE-like findings (total)	232/249 (93.2)
Polyarthritis	168/245 (68.6)
Lymphadenopathy	73/244 (29.9)
Facial erythema	57/245 (23.3)
Pericarditis or pleuritis	26/245 (10.6)
Leukopenia or thrombocytopenia	117/247 (47.4)
SSc-like findings (total)	218/249 (87.6)
Sclerodactyly	173/245 (70.6)
Interstitial lung disease	99/244 (40.6)
Oesophageal dysmotility or dilatation	40/232 (17.2)
IIM-like findings (total)	106/249 (42.6)
Muscle weakness	57/245 (23.3)
Elevated myogenic enzymes	80/239 (33.5)
Myogenic EMG findings	16/202 (7.9)
**Characteristic organ involvement**	
Pulmonary arterial hypertension	31/243 (12.8)
Aseptic meningitis	12/244 (4.9)
Trigeminal neuropathy	10/240 (4.2)

Abbreviations: EMG, electromyography; IIM idiopathic inflammatory myopathies; SLE, systemic lupus erythematosus; SSc, systemic sclerosis.

### Clinical phenotypes and overlap

We next examined phenotypic overlap based on combinations of SLE-like, SSc-like and IIM-like manifestations among 231 patients with sufficient data for all three categories (Fig. 1). Overlap between SLE-like and SSc-like features was the most common pattern, observed in 130 patients (56%). Triple overlap involving all three phenotypic domains was observed in 73 patients (32%). Less frequent patterns included SLE-like plus IIM-like features in 21 patients (9%), SLE-like features alone in 5 (2%) and SSc-like features alone in 2 (<1%), whereas isolated IIM-like features were not observed. In this context, polyarthritis was observed in 168 of 245 patients (68.6%). Among the 234 patients evaluable for joint damage, 13 (5.6%) demonstrated erosive changes, whereas 153 (65.4%) had non-erosive arthritis. In addition, pericarditis was observed in 18 of 242 patients (7.4%) and pleuritis in 17 of 244 patients (7.0%). Pulmonary function testing demonstrated a restrictive ventilatory impairment (%VC ≤ 80%) in 48 of 188 patients (25.5%) and reduced diffusing capacity (%DLCO ≤ 70%) in 52 of 184 patients (28.3%). Laboratory testing showed a median white blood cell count of 4600/µl (IQR, 3500–6300) and a median platelet count of 21.8 × 10^4^/µl (IQR, 16.2–27.3).

**Figure 1:**
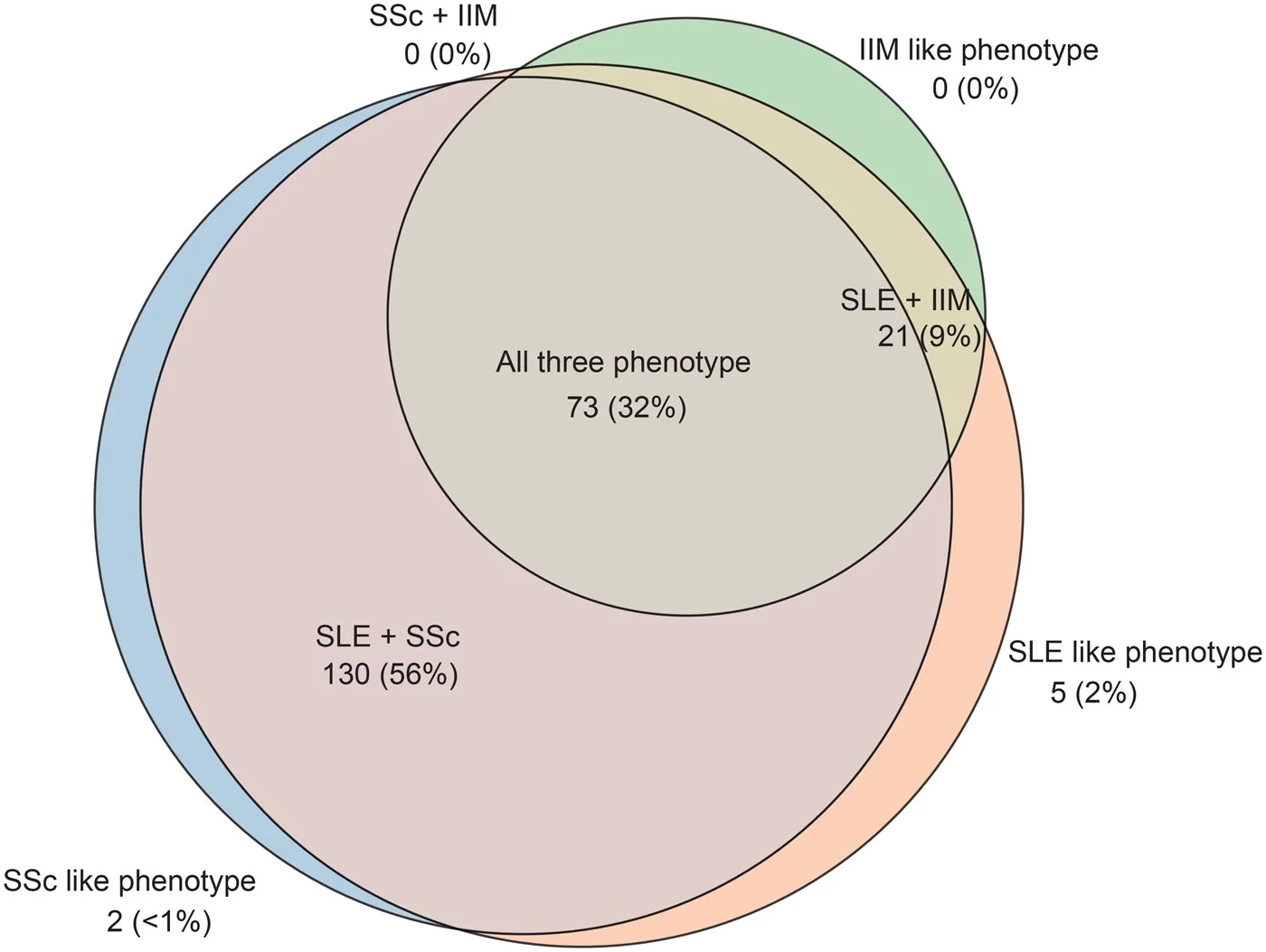
Overlap of clinical phenotypes in MCTD. An Euler diagram illustrating the overlap of SLE-like, SSc-like and IIM-like features among 231 evaluable patients who strictly fulfilled the diagnostic criteria. The area of each circle and intersection is proportional to the number of patients. The values indicate the number of patients (percentage)

With respect to autoantibodies other than anti-U1-RNP, antinuclear antibodies were detected in all tested patients (100%). Positivity rates for other autoantibodies were as follows: anti-dsDNA, 11.4% (27/237); anti-Sm, 13.9% (33/237); anti-topoisomerase I, 0.8% (2/237); anti-RNA polymerase III, 2.2% (2/92); anti-ARS including anti-Jo-1, 0.9% (1/112); and anti-CCP, 7.9% (11/139).

### Organ involvement and severity

Details of organ involvement and severity classification are summarized in Table 2. According to the MHLW severity classification, among 237 evaluable patients, 31 (13.1%) were categorized as mild, 138 (58.2%) as moderate and 68 (28.7%) as severe. Overall, at least one major organ complication was observed in 82 of 249 patients (32.9%). Major organ manifestations included pulmonary arterial hypertension in 31 of 243 patients (12.8%), advanced interstitial lung disease in 27 of 244 (11.1%), rapidly progressive interstitial lung disease in 14 of 244 (5.7%), thrombocytopenia in 20 of 247 (8.1%), aseptic meningitis in 12 of 244 (4.9%), haemolytic anaemia in 5 of 187 (2.7%), central nervous system manifestations in 7 of 237 (3.0%) and lower gastrointestinal dysfunction in 4 of 237 (1.7%). Patterns of co-occurrence of major organ manifestations are shown in Fig. 2. Severity distribution and organ involvement patterns in the broader cohort were comparable (Supplementary Table S4).

**Figure 2:**
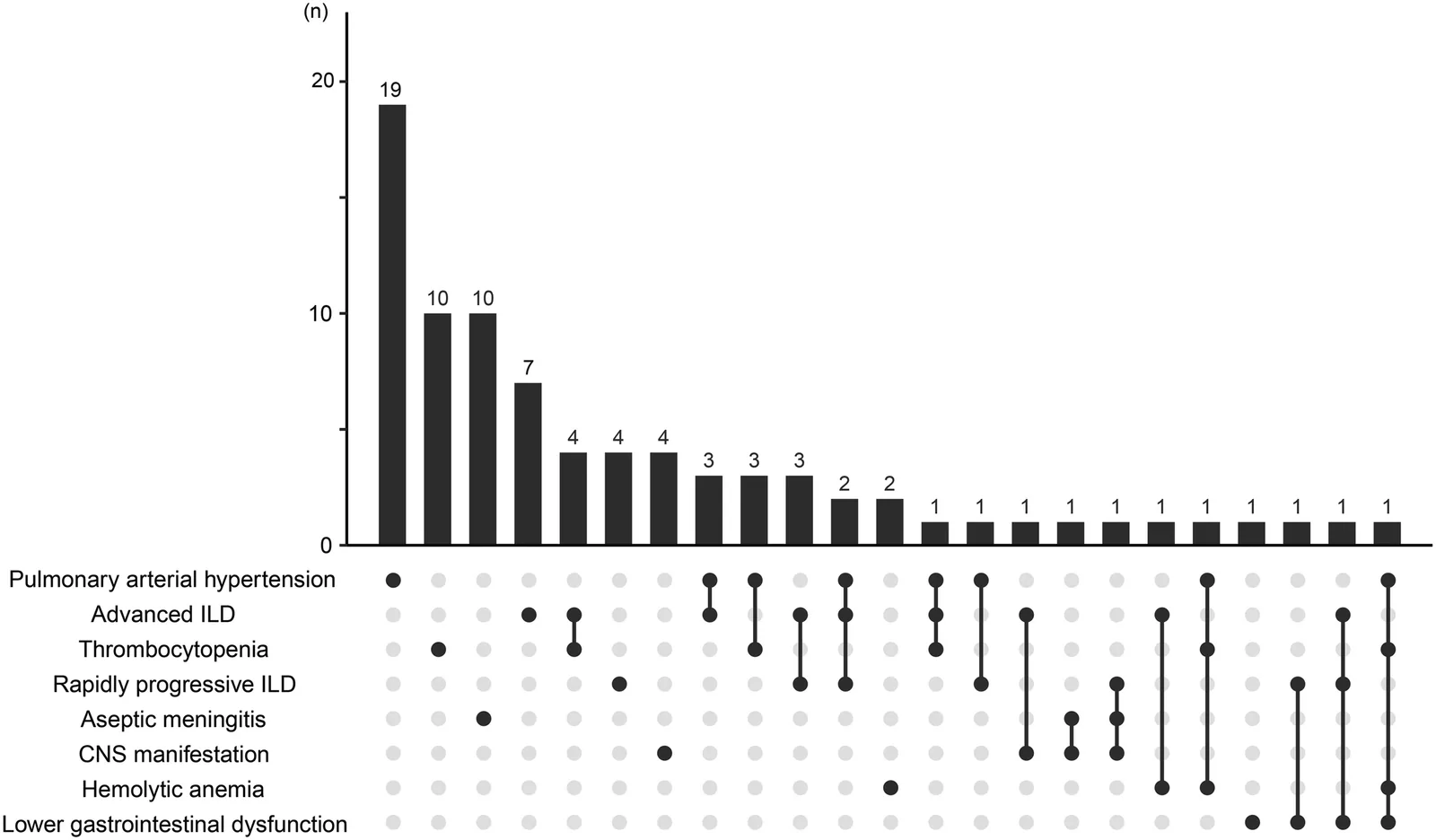
Patterns of co-occurrence of major organ involvements in MCTD. An UpSet plot illustrating the combinations of severe organ complications among 82 patients who exhibited at least one major organ involvement. The vertical bars indicate the number of patients for each specific combination of organ involvements. The horizontal bars represent the total number of patients presenting with each specific organ manifestation

**Table 2:** Disease severity classification and prevalence of major organ involvement.

Variables	*n*/*N* (%)
**Severity grading**	
Severe	68/237 (28.7)
Moderate	138/237 (58.2)
Mild	31/237 (13.1)
**Severe organ involvement**	
Pulmonary arterial hypertension	31/243 (12.8)
Advanced interstitial lung disease	27/244 (11.1)
Rapidly progressive interstitial lung disease	14/244 (5.7)
Thrombocytopenia	20/247 (8.1)
Aseptic meningitis	12/244 (4.9)
Haemolytic anaemia	5/187 (2.7)
Central nervous system manifestation	7/237 (3.0)
Lower gastrointestinal dysfunction	4/237 (1.7)

### Treatment patterns

Details of pharmacotherapy are summarized in Table 3. Glucocorticoids were administered in 162 of 243 evaluable patients (66.7%). Among GC-treated patients, the median maximum prednisolone-equivalent dose was 30.0 mg/day (IQR 15.0–50.0), and 96 of 156 (61.5%) received medium-to-high doses (≥30 mg/day). Intravenous glucocorticoid pulse therapy was administered in 37 of 153 GC-treated patients (24.2%). Concomitant immunomodulator/immunosuppressive therapy was administered in 134 of 237 patients (56.5%), most commonly hydroxychloroquine (19.0%), tacrolimus (13.1%), cyclophosphamide (11.0%) and methotrexate (10.5%). Biologics were used in 17 of 237 evaluable patients (7.2%), including rituximab in 7 patients, belimumab in 4 and tocilizumab and anifrolumab in 1 patient each. PAH-targeted therapies were prescribed in 36 of 238 patients (15.1%), whereas antifibrotic agents were used in 1 of 231 patients (0.4%). Treatment patterns in the broader cohort showed a similar profile (Supplementary Table S5).

**Table 3:** Treatment patterns and therapeutic regimens.

Variables	*n*/*N* (%)
**Glucocorticoids (GC)**	
GC use	162/243 (66.7)
Max dose (PSL equivalent), mg/day	30.0 [15.0–50.0]
High dose (≥30 mg/day)	96/156 (61.5)
Glucocorticoid pulse therapy (among GC users)	37/153 (24.2)
**Immunosuppressants**	
Any immunosuppressant use (≥1 agent)	134/237 (56.5)
Hydroxychloroquine	45/237 (19.0)
Tacrolimus	31/237 (13.1)
Cyclophosphamide	26/237 (11.0)
Methotrexate	25/237 (10.5)
Azathioprine	13/237 (5.5)
Ciclosporin	13/237 (5.5)
Mycophenolate mofetil	12/237 (5.1)
Biologics	
Any biologics use	17/237 (7.2)
Rituximab	7/237 (3.0)
Belimumab	4/237 (1.7)
Tocilizumab	1/237 (0.4)
Anifrolumab	1/237 (0.4)
Others	4/237 (1.7)
**Others**	
PAH-specific drugs (any)	36/238 (15.1)
Antifibrotic agents	1/231 (0.4)

Abbreviation: PAH, pulmonary arterial hypertension.

### Factors associated with disease severity

To identify baseline factors associated with severe classification, we compared patients in the Severe group (*n* = 68) with those in the Moderate/Mild group (*n* = 169) using variables that were not included in the MHLW severity definition (Table 4). There were no significant differences in sex (91.2% vs 91.7%, *P* = 1.000) or age at onset (median 47.4 vs 39.0 years, *P* = 0.052). Raynaud’s phenomenon was observed in 57 of 68 patients (83.8%) in the severe group and in 155 of 169 patients (91.7%) in the moderate/mild group (*P* = 0.165). The frequency of anti-U1-RNP antibody high titres did not differ between groups (23/68 [33.8%] vs 67/169 [39.6%], *P* = 0.492). Similarly, no significant between-group differences were observed for anti-dsDNA, anti-Sm or anti-topoisomerase I antibody positivity. Among the examined clinical manifestations, polyarthritis was less frequent in the Severe group than in the moderate/mild group (39/68 [57.4%] vs 122/169 [72.2%], *P* = 0.039). Conversely, manifestations such as lymphadenopathy, facial erythema, pericarditis or pleuritis, sclerodactyly, oesophageal dysmotility or dilatation, muscle weakness, elevated myogenic enzymes and myogenic EMG findings did not differ significantly between the groups. Furthermore, the overall prevalence of SLE-like, SSc-like and IIM-like findings did not significantly differ between the severity groups (*P* = 0.480, *P* = 0.340 and *P* = 1.000, respectively). These findings suggest that severe classification was not associated with anti-U1-RNP antibody high titres or with most baseline non-defining clinical features in this cohort. To further account for potential confounders, a multivariable logistic regression analysis was performed with severe disease classification as the dependent variable. Variables included in the model were anti-U1-RNP antibody high titre, age at onset, female sex, polyarthritis, Raynaud’s phenomenon and sclerodactyly. In this model, older age at onset was independently associated with severe disease (odds ratio [OR] 1.02, 95% CI 1.00–1.04; *P* = 0.041), whereas polyarthritis was inversely associated with severity (OR 0.54, 95% CI 0.29–1.00; *P* = 0.049). Anti-U1-RNP antibody off-scale-high status was not associated with severe classification after adjustment for these factors (OR 0.94, 95% CI 0.50–1.76; *P* = 0.853). The absence of an association between off-scale-high anti-U1-RNP antibody status and severe classification was also observed in the broader cohort (33.8% vs 38.5%, *P* = 0.592; Supplementary Table S6).

**Table 4:** Baseline characteristics and clinical manifestations stratified by severity grading (severe vs moderate/mild).

Variables	Moderate/Mild group (*n* = 169)	Severe group (*n* = 68)	*P*-value
**Demographics**			
Female sex	155 (91.7)	62 (91.2)	1
Age at onset, years, median [IQR]	39.0 [27.7–53.2]	47.4 [30.0–61.2]	0.052
Common findings			
Raynaud’s phenomenon	155 (91.7)	57 (83.8)	0.165
**Immunological findings**			
Anti-U1-RNP antibody positive	169 (100.0)	68 (100.0)	–
Anti-U1-RNP antibody high titre	67 (39.6)	23 (33.8)	0.492
Anti-dsDNA antibody	21 (12.4)	6 (8.8)	0.728
Anti-Sm antibody	21 (12.4)	12 (17.6)	0.496
Anti-topoisomerase I antibody	1 (0.6)	1 (1.5)	0.638
**Mixed findings**			
SLE-like findings (total)	160 (94.7)	62 (91.2)	0.480
Polyarthritis	122 (72.2)	39 (57.4)	0.039
Lymphadenopathy	53 (31.4)	18 (26.5)	0.242
Facial erythema	42 (24.9)	13 (19.1)	0.257
Pericarditis or Pleuritis	15 (8.9)	10 (14.7)	0.198
SSc-like findings (total)	151 (89.3)	57 (83.8)	0.340
Sclerodactyly	122 (72.2)	44 (64.7)	0.327
Oesophageal dysmotility or dilatation	27 (16.0)	13 (19.1)	0.140
IIM-like findings (total)	74 (43.8)	30 (44.1)	1
Muscle weakness	45 (26.6)	11 (16.2)	0.110
Elevated myogenic enzymes	54 (32.0)	24 (35.3)	0.736
Myogenic EMG findings	9 (5.3)	7 (10.3)	0.274

Abbreviations: EMG, electromyography; IIM idiopathic inflammatory myopathies; SLE, systemic lupus erythematosus; SSc, systemic sclerosis.

## Discussion

This multicentre study provides a comprehensive evaluation of clinical manifestations, major organ complications, real-world treatment patterns and their relationships to severity classification in a strictly defined cohort of 249 patients who fulfilled the 2019 MHLW diagnostic criteria for MCTD. Our cohort is one of the largest reported in Asia. Key patient characteristics, including female predominance (93.1%) and age at onset (median 40.6 years), were broadly comparable to those of the French cohort described by Chevalier *et al.* (88% female; median 35 years) [5]. The key findings of this study can be summarized as follows. First, among the phenotypic domains, our strictly defined cohort predominantly exhibited an overlap between SLE and SSc features (56.0%), with approximately one-third (32.0%) demonstrating concomitant findings across all three phenotypic domains (SLE, SSc and IIM). A recurrent critique regarding MCTD is whether these patients merely represent overlap syndromes of other established connective tissue diseases. However, our findings demonstrate that even when ambiguous clinical diagnoses are excluded and diagnostic criteria are strictly applied, the fundamental clinical reality of MCTD is characterized by these robust phenotypic overlaps. While some patients tested positive for anti-Sm (13.9%) or anti-dsDNA (11.4%) antibodies, the overall prevalence of SLE-, SSc- or IIM-like findings was not significantly associated with severity classification. This suggests that the clinical utility of the MCTD concept lies in identifying a high-risk group characterized by anti-U1-RNP seropositivity that requires multidisciplinary organ screening, rather than attempting to artificially isolate a ‘pure’ phenotype devoid of overlapping features. Second, to rigorously identify factors associated with severity classification, we explicitly excluded severity-defining criteria (such as ILD and cytopenias) from our comparative analysis to avoid circular reasoning. Through this approach, we demonstrated that broad phenotypic categories (SLE/SSc/IIM-like) and anti-U1-RNP antibody titres were not strongly associated with severity classification, although older age at onset was independently associated with severe disease. Severity classification appeared to be more closely linked to specific major organ complications than to the cumulative burden of broad overlapping phenotypic features. Third, although severe classification was largely linked to major organ complications, non-organ manifestations such as articular symptoms remained common in the cohort. These findings underscore that MCTD is not a uniform, fixed disease entity but encompasses substantial heterogeneity in both clinical phenotype and disease severity.

Previous reports have indicated that the presence of U1-RNP antibodies does not contribute to prognosis and have highlighted practical limitations in routine titre quantification, often omitting them from prognostic analyses [5, 17]. In this cohort, off-scale-high anti-U1-RNP antibody status, defined according to the assay-specific upper measurement limit, was not associated with severity classification after multivariable adjustment (OR 0.94, *P* = 0.853). However, anti-U1-RNP assays varied across institutions and calendar periods, and antibody results were therefore harmonized as off-scale high vs not off-scale high rather than analyzed as directly comparable quantitative titres. In addition, treatment status at the time of antibody measurement was not standardized and may have influenced antibody levels. Meanwhile, these results underscore the need for systematic, regular organ screening irrespective of serological levels [18–21].

Because no universally accepted diagnostic criteria for MCTD exist, the choice of diagnostic criteria used to define study cohorts is particularly relevant when interpreting cohort studies. Importantly, strict retrospective application of some historical MCTD criteria, including the original Sharp, Alarcón-Segovia and Kahn criteria, is challenging because they incorporate assay-specific serological requirements, including historical anti-RNP titre thresholds that cannot be directly mapped onto contemporary immunoassays. In the present study, we applied the 2019 MHLW criteria and found substantial concordance with the widely used Kasukawa criteria: 95.6% of patients fulfilling the MHLW criteria also fulfilled the Kasukawa criteria, with an overall agreement of 96.1% and a Cohen’s *κ* of 0.886. This high concordance indicates that the contemporary MHLW criteria largely identify the same clinical population as the conventional Kasukawa framework. Notably, all 11 patients who fulfilled the MHLW but not the Kasukawa criteria were classified through the characteristic organ involvement pathway, comprising PAH, aseptic meningitis or trigeminal neuropathy. Thus, the 2019 MHLW criteria preserve substantial continuity with the Kasukawa criteria while additionally capturing a small subgroup presenting with organ manifestations considered characteristic of MCTD.

Regarding the MHLW severity classification, ILD was documented in ∼40% of the cohort (99/244, 40.6%), and PAH was present in 12.8% of evaluable patients (31/243). These major organ complications have a substantial impact on patients’ QOL and are well recognized as key determinants of MCTD and related connective tissue diseases [22, 23]. However, the MHLW severity classification should be interpreted in the context of its intended use. It was not developed as a prognostic score but a practical clinical framework used nationwide in Japan as part of the eligibility assessment for public medical expense subsidies for designated intractable diseases. Accordingly, the present study describes the clinical distribution and correlates of this real-world severity framework rather than validating its prognostic performance. Non-organ manifestations also remained common across the cohort. Although the current severity framework primarily emphasizes major organ involvement, manifestations such as arthritis and sclerodactyly may substantially compromise activities of daily living and QOL, highlighting the need for therapeutic targets that address pain, function and disability independently of conventional severity grading [24].

In terms of treatment patterns, glucocorticoids remained the mainstay, and high-dose regimens, pulse therapy and combination immunosuppression were frequently employed. In contrast, biologic use was limited. This likely reflects the paucity of MCTD-specific evidence and the pragmatic challenges of routine care, where therapeutic indications must be individualized according to the dominant overlapping phenotype. In practice, clinicians appear to extrapolate treatment algorithms from SLE, SSc or IIM rather than from MCTD-specific trials, underscoring the urgent need for prospective studies and consensus-based treatment recommendations tailored to MCTD [25].

This study has several limitations. First, as a retrospective analysis based on standardized clinical survey forms, it is susceptible to inter-facility variability in assessment and incomplete data capture. To address diagnostic ambiguity, we restricted our primary analysis to patients strictly fulfilling the classification criteria. However, sensitivity analyses including the broader clinically diagnosed cohort (*n* = 311) showed comparable patterns of clinical phenotype, organ involvement, treatment and the absence of association between anti-U1-RNP antibody titres and disease severity (Supplementary Tables S2 and S4–S6). Second, because the cohort was derived from university hospitals, referral bias may have enriched the sample for more severe disease. Third, the cross-sectional nature of the present analysis precludes evaluation of temporal changes in disease activity, treatment response and long-term outcomes, including survival. Therefore, the prognostic validity of the MHLW severity classification could not be assessed in this study and requires validation in a longitudinal cohort with systematically collected follow-up and survival data. Furthermore, as this registry utilized historical data, PAH was diagnosed based on prevailing standards at the time (e.g. the 2008 World Symposium criteria). Because the diagnostic threshold for mean pulmonary arterial pressure was lowered to >20 mmHg in the 2022 ESC/ERS guidelines, the true prevalence of PAH in our cohort may be underestimated compared with contemporary standards [21]. In addition, the MHLW definition of rapidly progressive ILD does not include an explicit temporal threshold, and its retrospective classification therefore depended partly on physician judgement. Fourth, autoantibody measurements and imaging assessments were not fully standardized across centres, which may have introduced additional heterogeneity into the dataset. Long-term outcomes in patients who do not fully satisfy diagnostic criteria, as well as the trajectory of organ involvement following therapeutic interventions, are currently under investigation.

In conclusion, MCTD in routine clinical practice displays marked heterogeneity in both clinical phenotype and disease severity. These findings reaffirm the need for systematic screening, focusing on cardiopulmonary involvement, and for individualized management tailored to the predominant clinical (SLE-, SSc- or IIM-like) phenotype. Dedicated studies using standardized quantitative assays, preferably with pretreatment samples and longitudinal clinical outcomes, are required to determine the clinical utility of anti-U1-RNP antibody titres.

## Supplementary Material

keag577_Supplementary_Data

## Data Availability

The datasets generated during and/or analyzed during the current study are available from the corresponding author on reasonable request.
