# Supplementary material for: Clinical characteristics and severity classification in mixed connective tissue disease: a nationwide retrospective study
Source: Rheumatology (Oxford). 2026 Oct 15;65(10):keag577. doi: 10.1093/rheumatology/keag577 (PMC13693593; doi:10.1093/rheumatology/keag577)
Supplement: Supplementary file 1 [file keag577_supplementary_data.zip › rhe-26-1229-File004.docx]

Supplemental Table S1. Disease severity classification for MCTD defined by the Ministry of Health, Labour and Welfare

| Severity | Organ Involvement | Clinical Findings |
| --- | --- | --- |
| **Severe** | (Presence of 1 or more of the following) |  |
|  | Central Nervous System Symptoms | Seizures, organic brain syndrome, psychosis, cerebrovascular disorder |
|  | Aseptic Meningitis | Headache, nausea, vomiting |
|  | Pulmonary Arterial Hypertension | Shortness of breath, palpitations, retrosternal pain |
|  | Rapidly Progressive Interstitial Lung Disease | Rapidly progressive dyspnea, cough |
|  | Advanced Interstitial Lung Disease | Palpitations, shortness of breath, cough |
|  | Thrombocytopenia | Bleeding tendency, purpura |
|  | Hemolytic Anemia | Severe anemia |
|  | Lower Gastrointestinal Dysfunction | Malabsorption syndrome, intestinal pseudo obstruction |
| **Moderate** | (Presence of 1 or more of the following) |  |
|  | Fever | Observed during periods of high disease activity |
|  | Lymphadenopathy | Observed during periods of high disease activity |
|  | Myositis | Muscle weakness, myalgia, elevated myogenic enzymes. |
|  | Esophageal Dysmotility | Reflux esophagitis, heartburn, epigastric pain |
|  | Serositis | Pleural effusion, pericardial effusion |
|  | Renal Involvement | Proteinuria |
|  | Cutaneous Vasculitis | Purpura, nail bed hemorrhage, skin infarction |
|  | Skin Ulcers / Gangrene | Due to severe peripheral circulatory disturbance |
|  | Interstitial Lung Disease | Progression is usually slow |
|  | Peripheral Neuropathy | Trigeminal neuralgia is common |
|  | Erosive / Destructive Arthritis | Joint destruction is sometimes seen |
| **Mild** | (Presence of 1 or more of the following) |  |
|  | Raynaud's Phenomenon | Color changes in fingers due to vasospasm from cold stimuli. |
|  | Swelling of fingers or back of hands | Important for MCTD diagnosis but clinically not problematic |
|  | Erythema | Often on the face, palms, etc. |
|  | Sclerodactyly (Limited to fingers) | Remains mild, but can lead to flexion contractures of the fingers |
|  | Non-destructive Arthritis | Joint destruction is usually not present but is sometimes seen |

Supplemental Table S2: Demographics & clinical manifestations of the broader clinically diagnosed cohort (n=311)

| Variables | n/N (%) |
| --- | --- |
| Demographics |  |
| Female sex | 286/307 (93.2) |
| Age at onset, years | 40.6 [28.6-55.6] |
| Family history of MCTD | 22/241 (9.1) |
| Common Findings |  |
| Raynaud's phenomenon or swollen fingers/hands | 275/285 (96.5) |
| Raynaud's phenomenon | 247/269 (91.8) |
| Immunological Findings |  |
| Anti-U1-RNP antibody positive | 286/286 (100.0) |
| Mixed Findings (Organ Involvement) |  |
| SLE-like findings (Total) | 256/287 (89.2) |
| Polyarthritis | 186/281 (66.2) |
| Lymphadenopathy | 80/277 (28.9) |
| Facial erythema | 65/278 (23.4) |
| Pericarditis or Pleuritis | 28/280 (10.0) |
| Leukopenia or Thrombocytopenia | 123/279 (44.1) |
| SSc-like findings (Total) | 236/284 (83.1) |
| Sclerodactyly | 184/277 (66.4) |
| Interstitial lung disease | 109/279 (39.1) |
| Esophageal dysmotility or dilatation | 43/266 (16.2) |
| IIM-like findings (Total) | 108/283 (38.2) |
| Muscle weakness | 58/280 (20.7) |
| Elevated myogenic enzymes | 82/271 (30.3) |
| Myogenic EMG findings | 16/221 (7.2) |
| Characteristic Organ Involvement |  |
| Pulmonary arterial hypertension | 31/282 (11.0) |
| Aseptic meningitis | 12/281 (4.3) |
| Trigeminal neuropathy | 11/273 (4.0) |

SLE, systemic lupus erythematosus; SSc, systemic sclerosis; IIM idiopathic inflammatory myopathies; EMG, electromyography.

Supplemental Table S3: Concordance between the 2019 MHLW and Kasukawa diagnostic criteria in the broader clinically diagnosed MCTD cohort

Part A. Concordance between criteria

|  | Kasukawa fulfilled | Kasukawa not fulfilled | Total |
| --- | --- | --- | --- |
| MHLW 2019 fulfilled | 238 | 11 | 249 |
| MHLW 2019 not fulfilled | 1 | 61 | 62 |
| Total | 239 | 72 | 311 |

Overall agreement 299/311 (96.1%), Cohen’s κ 0.886, 95% CI 0.819–0.944

MHLW 2019 fulfilled who also fulfilled Kasukawa 238/249 (95.6%)

Kasukawa fulfilled who also fulfilled MHLW 2019 238/239 (99.6%)

Part B. Reasons for discordance

| Discordant group | n | Explanation |
| --- | --- | --- |
| MHLW 2019 only | 11 | All patients fulfilled the characteristic organ involvement pathway (PAH, n=6; aseptic meningitis, n=4; trigeminal neuropathy, n=1) but did not fulfill the Kasukawa requirement for mixed findings in at least two disease categories. |
| Kasukawa only | 1 | The SSc-like domain was fulfilled because of reduced DLCO, despite the absence of ILD. |

Supplemental Table S4: Severity classification & organ involvement in n=311

| Variables | n/N (%) |
| --- | --- |
| Severity Grading |  |
| Severe | 68/263 (25.9) |
| Moderate | 154/263 (58.6) |
| Mild | 41/263 (15.6) |
| Severe Organ Involvement |  |
| Pulmonary arterial hypertension | 31/282 (11.0) |
| Advanced interstitial lung disease | 27/267 (10.1) |
| Rapidly progressive interstitial lung disease | 14/265 (5.3) |
| Thrombocytopenia | 20/264 (7.6) |
| Aseptic meningitis | 12/281 (4.3) |
| Hemolytic anemia | 6/210 (2.9) |
| Central nervous system manifestation | 7/259 (2.7) |
| Lower gastrointestinal dysfunction | 4/259 (1.5) |

Supplemental Table S5: Treatment patterns in n=311

| Variables | n/N (%) |
| --- | --- |
| Glucocorticoids (GC) |  |
| GC use | 184/281 (65.5) |
| Max dose (PSL equivalent), mg/day | 30.0 [14.4-50.0] |
| High dose (≥30 mg/day) | 106/177 (59.9) |
| Glucocorticoid pulse therapy (among GC users) | 38/175 (21.7) |
| Immunosuppressants |  |
| Any immunosuppressant use (≥1 agent) | 146/275 (53.1) |
| Hydroxychloroquine | 46/275 (16.7) |
| Tacrolimus | 34/275 (12.4) |
| Cyclophosphamide | 28/275 (10.2) |
| Methotrexate | 29/275 (10.5) |
| Azathioprine | 14/275 (5.1) |
| Cyclosporine | 13/275 (4.7) |
| Mycophenolate mofetil | 13/275 (4.7) |
| Biologics |  |
| Any biologics use | 20/272 (7.4) |
| Rituximab | 7/272 (2.6) |
| Belimumab | 4/272 (1.5) |
| Tocilizumab | 4/272 (1.5) |
| Anifrolumab | 1/272 (0.4) |
| Others | 4/272 (1.5) |
| Others |  |
| PAH-specific drugs (any) | 42/273 (15.4) |
| Antifibrotic agents | 2/265 (0.8) |

PAH, pulmonary arterial hypertension.

Supplemental Table S6: Severity comparison (Severe vs Moderate/Mild) in n=311

| Variables | Moderate/Mild group  (n=195) | Severe group  (n=68) | p-value |
| --- | --- | --- | --- |
| Demographics |  |  |  |
| Female sex | 179 (91.8) | 62 (91.2) | 1 |
| Age at onset, years, median [IQR] | 40.5 [28.2-53.7] | 47.4 [30.0-61.2] | 0.082 |
| Common Findings |  |  |  |
| Raynaud's phenomenon or swollen fingers/hands | 187 (95.9) | 68 (100.0) | 0.198 |
| Raynaud's phenomenon | 172 (88.2) | 57 (83.8) | 0.473 |
| Immunological Findings |  |  |  |
| Anti-U1-RNP antibody positive | 195 (100.0) | 68 (100.0) | - |
| Anti-U1-RNP antibody high titer | **75 (38.5)** | **23 (33.8)** | **0.592** |
| Anti-dsDNA antibody | 23 (11.8) | 6 (8.8) | 0.654 |
| Anti-Sm antibody | 23 (11.8) | 12 (17.6) | 0.310 |
| Anti-Topoisomerase I antibody | 2 (1.0) | 1 (1.5) | 1.000 |
| Mixed Findings (Organ Involvement) |  |  |  |
| SLE-like findings (Total) | 174 (89.2) | 62 (91.2) | 0.823 |
| Polyarthritis | 133 (68.2) | 39 (57.4) | 0.141 |
| Lymphadenopathy | 58 (29.7) | 18 (26.5) | 0.721 |
| Facial erythema | 47 (24.1) | 13 (19.1) | 0.499 |
| Pericarditis or Pleuritis | 15 (7.7) | 10 (14.7) | 0.145 |
| Leukopenia or Thrombocytopenia | 78 (40.0) | 37 (54.4) | 0.055 |
| SSc-like findings (Total) | 165 (84.6) | 57 (83.8) | 1.000 |
| Sclerodactyly | 133 (68.2) | 44 (64.7) | 0.704 |
| Interstitial lung disease | 65 (33.3) | 37 (54.4) | **0.003** |
| Esophageal dysmotility or dilatation | 29 (14.9) | 13 (19.1) | 0.528 |
| IIM-like findings (Total) | 76 (39.0) | 30 (44.1) | 0.548 |
| Muscle weakness | 46 (23.6) | 11 (16.2) | 0.268 |
| Elevated myogenic enzymes | 56 (28.7) | 24 (35.3) | 0.389 |
| Myogenic EMG findings | 9 (4.6) | 7 (10.3) | 0.164 |

SLE, systemic lupus erythematosus; SSc, systemic sclerosis; IIM idiopathic inflammatory myopathies; EMG, electromyography.
